# Ectopic ovary with torsion: uncommon diagnosis made by ultrasound

**DOI:** 10.1590/0100-3984.2014.0031

**Published:** 2017

**Authors:** Adham do Amaral e Castro, Fernando Morandini, Caroline Paludo Calixto, Wagner Haese Barros, Edson Tetsuya Nakatani, Allan do Amaral e Castro

**Affiliations:** 1 MSc, Doctoral Student at the Medical Research Institute of the Faculdade Evangélica do Paraná - Hospital Evangélico de Curitiba, Curitiba, PR, Postgraduate Student in Musculoskeletal Radiology at the Hospital Israelita Albert Einstein, São Paulo, SP, Brazil.; 2 MD, Radiologist at Hospital Vita Curitiba, Curitiba, PR, Brazil.; 3 MD, Resident in Radiology and Diagnostic Imaging at the Hospital Universitário Evangélico de Curitiba, Curitiba, PR, Brazil.; 4 MD, Radiologist for the Grupo Fleury / Hospital Alemão Oswaldo Cruz, São Paulo, SP, Brazil.; 5 MD, Gynecologist and Obstetrician, Specialist in Fetal Medicine at the Instituto da Mulher e Medicina Fetal, Curitiba, PR, Brazil.; 6 MD, Radiologist at Prefeitura Municipal de Paranaguá, Paranaguá, PR, Brazil.

**Keywords:** Ectopic ovary, Ultrasonography, Ovary torsion

## Abstract

Ultrasound is an important diagnostic tool in inguinal hernia and in the evaluation of the contents of the hernia sac. This report presents a case in which ultrasound revealed a herniated ectopic ovary, complicated by torsion of its vascular pedicle, in the right labia majora. We also present a brief discussion of ovarian hernia, its potential complications, and the treatments available.

## Abstract (pt)

A ultrassonografia é uma importante ferramenta diagnóstica no quadro de hérnia inguinal e na avaliação do conteúdo do saco herniário. Neste relato será apresentado um caso com diagnóstico ultrassonográfico de ovário ectópico herniado na região do grande lábio vulvar direito, complicado com torção de seu pedículo vascular, assim como será feita uma breve discussão sobre herniação ovariana, suas complicações e tratamentos.

## INTRODUCTION

The differential diagnosis of a labial mass in a prepubescent patient includes inguinal hernia, ultrasound being an important tool in the diagnosis and evaluation of the contents of the hernia sac^(1)^. Here, we report a case of herniation of the right ovary through the ipsilateral labia majora, complicated by ovarian torsion.

## CASE REPORT

A two-month-old female patient was brought to the emergency department of our hospital by her mother, who reported a "painful lump" on the right labia majora of the infant. On physical examination, we observed palpable, irreducible, localized nodulation, with no signs of inflammation.

**Figure 1:**
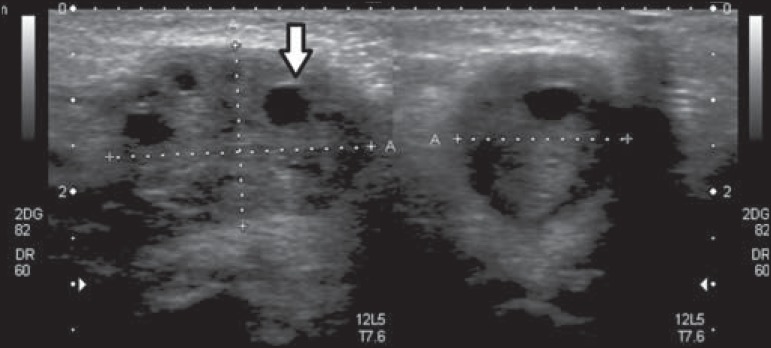
Ultrasound of the right labia majora. Note the right ovary, which is enlarged, located on the ipsilateral labia majora and the multiple peripheral cysts (arrow).

**Figure 2:**
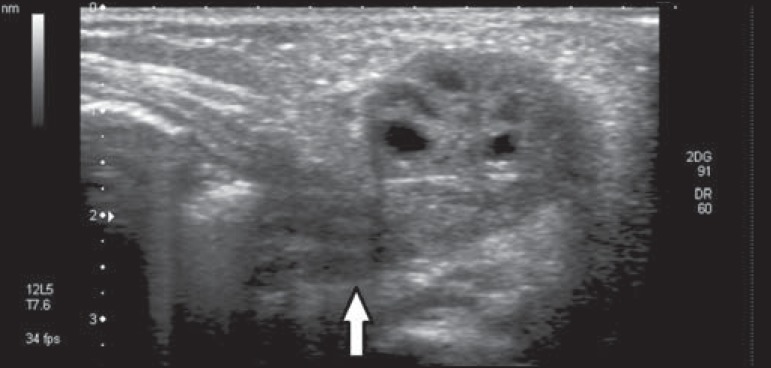
Ultrasound of the right labia majora. Note the invasion of the pedicle into the inguinal canal (arrow).

**Figure 3:**
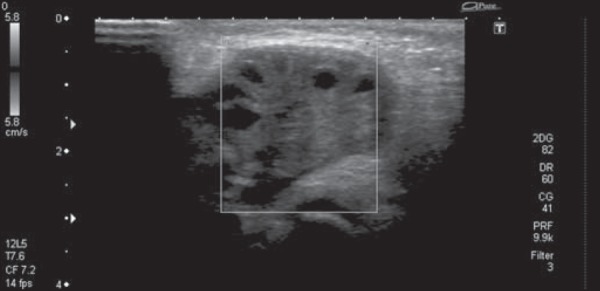
Doppler flow study showing the absence of vascular flow in the right ovary.

Ultrasound revealed a right-sided solid structure with multiple peripheral cysts, characterized as an ectopic ovary (Figures 1 and 2). On Doppler flow studies, we observed no ovarian flow. That finding, together with the multiple peripheral cysts and the increased size of the organ, suggested the diagnosis of torsion (Figure 3).

**Figure 4:**
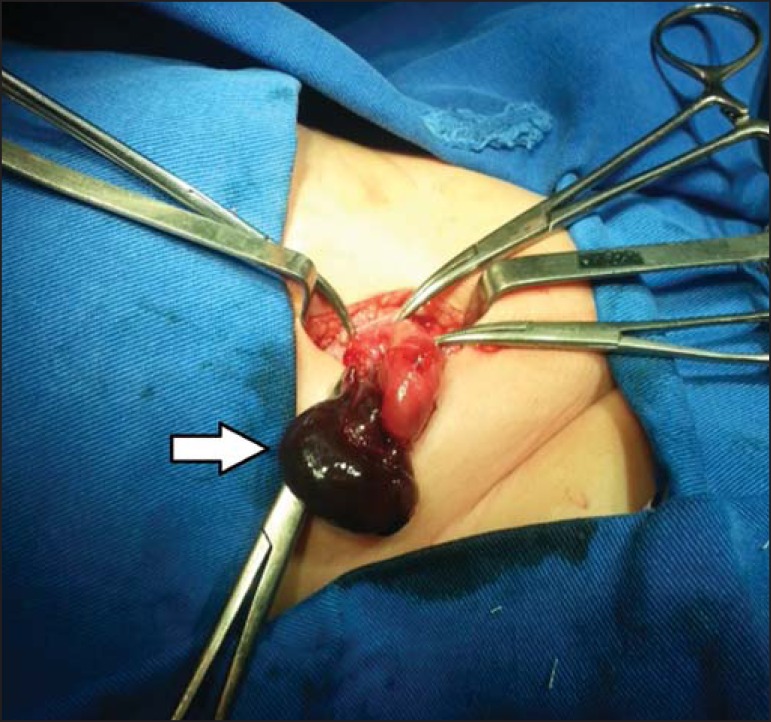
Signs of ischemia in the right ovary (arrow) identified during surgery.

We carried out emergency surgery, which revealed signs of ischemia in the affected ovary (Figure 4). After reversal of the torsion, the ischemia diminished, the ovary was repositioned within the abdominal cavity, connecting to the hernia sac. The patient showed favorable postoperative evolution.

## DISCUSSION

The formation of the inguinal canal is centered in the *gubernaculum testis* and the *processus vaginalis*^(2)^. The first is a fibromuscular cord, which, in girls, gives rise to the labia majora and the inferior pole of the fetal gonad. The *gubernaculum testis* also gives rise to the uterine-ovarian ligament and the round ligament, which connect the ovary to the medial region of the uterus, preventing its descent via the inguinal canal^(2,3)^. The *processus vaginalis peritonei* (also known as the canal of Nuck in females) is a tubular invagination (pouch) within the inguinal canal. Normally, it is completely obliterated in females^(2)^. In rare cases, failure of the obliteration process leads to congenital alterations, such as evagination of the canal of Nuck in the inguinal canal or in the labia majora^(3)^.

Herniation of the canal of Nuck involves the ovary in 15-20% of cases^(4)^, increasing the risk of the ovary becoming trapped or strangulated, because it becomes swollen to the extent that it is trapped, progressively increasing in volume and becoming less compressible than bowel loops^(5)^. The risk of torsion also increases when there is suspension and narrowing of the vascular pedicle of the ovary^(6)^.

The choice between elective and emergency surgery in the treatment of a trapped ovarian hernia using either is controversial. In cases of strangulation or torsion, emergency surgery is preferable. The surgical technique involves exposure of the peritoneal sac for evaluation of its content, the viscera being returned to the abdominal cavity when viable, with high ligation of the hernia sac. If the vitality of the hernia content is impaired, warm compresses are applied for a few minutes in order to evaluate the degree of improvement and thus determine the best practice; in the absence of improvement, the viscera should be resected^(7)^.

Typical ovarian morphology can be observed on ultrasound, and blood flow can be identified through Doppler flow studies^(8)^.

This report demonstrates the importance of the use of ultrasound in the diagnosis of ovarian torsion in herniation of the canal of Nuck. In the case presented here, the condition was confirmed and treated through surgery, which permitted the preservation of the organ.
